# Structure of a Conserved RNA Element in the SARS Virus Genome Determined

**DOI:** 10.1371/journal.pbio.0030029

**Published:** 2004-12-28

In February 2003, the first (and so far only) epidemic of severe acute respiratory syndrome (SARS) started in Guangdong Province, China. A respiratory illness that begins with a high temperature and can develop into life-threatening pneumonia, SARS is spread by close person-to-person contact. Before the end of the month, a Guangdong doctor had inadvertently taken the infection to Hong Kong. A woman staying in the same Hong Kong hotel as the doctor then carried the disease to Toronto. In March, the World Health Organization issued a global alert and warned against unnecessary travel to affected areas. Because of these and other containment efforts, 8,098 people became ill with SARS, rather than the predicted millions; 774 people died. The last case of the epidemic was reported in Taiwan in June 2003, and since then there have been only two cases in Singapore and nine in China.

By May 2003, a coronavirus had been identified as the cause of SARS, and the full genome sequence of this new human pathogen, which may have jumped from civet cats to people, had been published. From the viral genome, researchers have deduced the sequences and structures of the viral proteins, hoping to use this information to develop treatments and vaccines for SARS. But could the structure of the RNA genome itself also be a target for antiviral drugs?[Fig pbio-0030029-g001]

**Figure pbio-0030029-g001:**
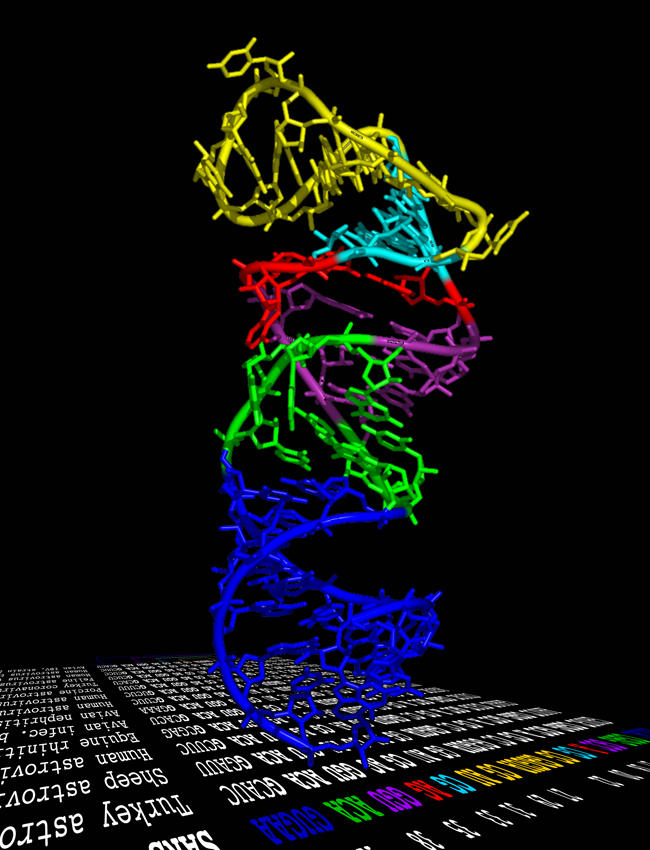
Structure of a conserved RNA element within the SARS virus genome

The genome of the SARS virus is a single strand of RNA that folds into regular repeating patterns to form secondary structures such as helices. These then fold and bend in three dimensions to form complex tertiary structures. William Scott and colleagues have used X-ray crystallography to measure the exact positions of individual ribonucleotides and the interactions between them in a small segment of the SARS virus genome called the s2m element. This element sits at one end of the viral genome, and, as the researchers show, its sequence is highly conserved in related coronaviruses. Furthermore, unlike the rest of the SARS genome, which changes rapidly, the s2m element is absolutely conserved in SARS variants obtained from patients during the SARS epidemic. This strong sequence conservation indicates that the tertiary structure of s2m could be important for viral function, and when the researchers solved the three-dimensional crystal structure of the element, they found that it had a unique tertiary structure. In particular, there was a right-angle kink in its helical axis and a tunnel with a net negative charge.

The biological role of a new protein can often be deduced by comparing its shape with that of proteins with known functions. Scott and colleagues used this approach to hypothesize that the function of the s2m element involves interaction with a conserved host factor during the SARS life cycle. Finding a similar 90° kink in a region of ribosomal RNA that binds factors necessary for the initiation of protein synthesis, the researchers speculate that the SARS virus may use the s2m element to hijack its host cell's protein synthesis machinery. This and other putative roles need to be tested experimentally, but given that the s2m element is absent in the human genome, its unusual structural features could be an attractive target for the design of antiviral therapeutic agents.

